# Women Resident Doctors in Poor-Law Infirmaries

**Published:** 1912-09-07

**Authors:** Catherine Kirk

**Affiliations:** Resident Assistant Medical Officer, Poor-Law Hospital, Halifax.


### WOMEN RESIDENT DOCTORS IN POOR-LAW INFIRMARIES.

By CATHERINE KIRK, M.A., M.B., Resident Assistant Medical Officer, Poor-Law Hospital, Halifax.

I have been asked to write upon the question of women *versus* men doctors for Poor-Law hospitals. The points I have been requested to consider are generally as follows:—(*a*) Have you found in your own experience many or any cases with which a woman doctor could not properly deal? (*b*) What is the usual experience of other resident women doctors? (*c*) Do you think women well fitted for the administration attached to the posts? (*d*) And any reasons why, in your judgment, they may be preferable to men?

I have lettered these points and will answer them accordingly.

St. Luke's Hospital is a Poor-Law institution of 400 beds, built ten or eleven years ago. There are two visiting medical officers, who attend on alternate days. In my experience of eighteen months in the capacity of sole resident here there has not been a medical or surgical case with which a woman doctor could not deal. I suppose the method of receiving patients into hospital is the same here as in other similar Poor-Law institutions—viz., the patient brings with him two certificates, one from the relieving-officer and the other from the parish doctor of his own district. These certificates bear, besides other particulars, a diagnosis of the case. As resident I examine every patient admitted, enter name, etc., into the admission book, and affix the number of the ward to which the patient is to go. Now in cases of venereal disease in male patients—the diagnosis being already made—one would accept it and send the patient to the proper ward, where the male attendant in charge bathes and examines him. These, therefore, are the cases which I leave to one or other of the visiting surgeons according as the case is allotted to them. Their attention is drawn to these cases (as to all new cases), and they are examined. Of course, I always treat their general condition and, when " Salvarsan " is prescribed, give the injections.

I have met three women doctors who have been resident at this hospital, and their experiences have been similar to mine. One of these was the first woman resident (seven years ago), and she stayed two years. I think I am right when I say that she is most decidedly in favour of women residents for workhouse infirmaries.

I cannot myself quite understand the continued prejudice towards women residents in hospitals. As I go round the wards, the patients on the male side are just as much my patients as those on the female side. In fact some of the most varied and interesting cases are amongst the former in both acute and chronic diseases. It is foolish and ridiculous for people to allow that a woman can nurse a man perfectly but still to repudiate the idea of a woman doctoring a man. Why, indeed, are there women nurses for men if the work is thought indelicate? Everyone knows that women nurses are indispensable, and no right-minded man would resent a woman nursing him were he suffering from pneumonia, acute rheumatism, appendicitis, or a fractured thigh. Why, then, should he recoil from the suggestion that a medical woman should prescribe for him (or for his poorer neighbours in a hospital) during such illnesses? A nurse has infinitely more to *do for* the patient than the doctor. As regards administration, I think most women doctors are admirably fitted for that work.

I do not know that I have any special reasons for saying that in my opinion women are preferable to men; for my own part I cannot see why there should be any difference, the one may be as good as the other in the matter of ability and experience. Boards of Guardians and hospital committees who have engaged women to be their residents should be able to give impartial judgment. As far as information is gathered from them, one finds that the opinion holds that men as residents to Poor-Law hospitals are inclined to be slack in their duties; that under women residents " the thing works remarkably well." I think I do not exaggerate when I say that I feel sure the Halifax Board of Guardians never mean to contemplate a return to men residents. I may add that most of the medical men with whom I have had acquaintance agree in their opinion that women are certainly more conscientious and more thorough in their work. Perhaps the Poor-Law Guardians of England and the Parish Councillors of Scotland have begun to discover this and act accordingly. I trust that in the near future woman's worth may be revealed in the matter of acting as a responsible house surgeon and in that of medical superintendent to a hospital.

